# The use of routinely collected structural neuroimaging to identify cognitive impairment in multiple sclerosis

**DOI:** 10.1186/s12883-026-05085-z

**Published:** 2026-06-17

**Authors:** Reine E. Jardine, Valeriya Kuznetsova, Fiore D’Aprano, Tomas Kalinicik, Stefanie Roberts, Charles B. Malpas

**Affiliations:** 1 Melbourne School of Psychological Sciences, The University of Melbourne, Redmond Barry Building, Parkville, VIC 3010 Australia; 2 Neuroimmunology Centre, Department of Neurology, The Royal Melbourne Hospital, 635 Elizabeth Street, Melbourne, VIC 3000 Australia; 3 Clinical Outcomes Research (CORe) Unit, Department of Medicine (RMH), The University of Melbourne, 635 Elizabeth Street, Melbourne, VIC 3000 Australia; 4 Centre of Excellence for Cellular Immunotherapy and Clinical Haematology, Peter MacCallum Cancer Centre and Royal Melbourne Hospital, 305 Grattan Street, Melbourne, VIC 3000 Australia; 5 School of Psychology, Deakin University, Victoria, Australia

**Keywords:** Multiple sclerosis, Cognitive impairment, Structural neuroimaging markers, Screening, Clinical data

## Abstract

**Background:**

Cognitive impairment is common in multiple sclerosis (MS), yet comprehensive cognitive assessment is not universally accessible, and patients need to be triaged for referral. Given the links between brain atrophy and cognition, this study investigated whether routinely collected neuroimaging markers could identify MS patients at risk of cognitive impairment.

**Methods:**

Data were retrospectively analysed from adult MS patients assessed in a specialist cognitive neuroimmunology clinic, who had undergone MRI within 12 months prior to cognitive testing. Normalised brain volume (NBV), normalised grey matter volume (NGMV), normalised white matter volume (NWMV), and corpus callosum index (CCI) were measured using the Siemens MorphoBox automated software. GLMs were estimated to investigate group differences in brain volume metrics between cognitively impaired and non-impaired patients. ROC curves were estimated to investigate screening performance for neuroimaging metrics (Youden’s J). Results are expressed as parameter estimates with 95% bootstrapped confidence intervals (CI).

**Results:**

120 patients were included (35% cognitively impaired). Cognitively impaired patients had lower NBV (*b* = − 2.06, 95% CI [− 3.35, − 0.81]) and NWMV (*b* = − 1.65, 95% CI [− 2.60, − 0.77]). NWMV (area under the curve [AUC] = 0.67, 95% CI [0.57, 0.76]) and CCI (AUC = 0.62, 95% CI [0.51, 0.72]) classified impairment, although sensitivity was low (< 0.70). No clear associations or sufficient classification performance were observed for NGMV. Diagnostic performance improved when neuroimaging markers were statistically combined with relevant demographic information.

**Conclusion:**

The present study did not find strong evidence supporting routinely collected neuroimaging as standalone cognitive screening tools. Classification performance improved when combined with demographic factors, but remained below thresholds for clinical utility. These findings highlight a gap between group-level associations reported in the literature and their translation to individual-level clinical application.

**Supplementary Information:**

The online version contains supplementary material available at 10.1186/s12883-026-05085-z.

## Introduction

With an estimated prevalence of approximately 29–36%, cognitive impairment is a disabling consequence of multiple sclerosis (MS) [1, 2]. Access to routine specialist cognitive examination, however, is often challenging in clinical practice due to the associated time and cost constraints [3]. There is a need to efficiently identify patients who are at increased risk of cognitive impairment to accurately triage referral and maximise opportunities for cognitive intervention [4]. Given that MS is associated with brain atrophy in regions and networks involved in cognition, structural magnetic resonance imaging (MRI) could be a promising non-invasive proxy for cognitive function [5]. If effective, this approach could provide a low-burden, cost-effective means of screening for cognitive risk, leveraging routinely collected MRI data that is already integrated into standard MS care [6].

Research investigating MRI-based markers of cognition in MS has primarily focused on global metrics, such as normalised brain volume (NBV), a measure of overall brain volume corrected for normal variation in head size [7]. This focus is based on the premise that MS is characterised by inflammatory neurodegenerative processes which lead to cumulative burden of lesions, plaques, and diffuse tissue injury contributing to measurable brain volume changes, making brain volume a marker of interest in studies of cognition in MS [8]. These investigations, which often include tissue-specific brain volume measures, have also shown that measures such as normalised grey matter volume (NGMV) and normalised white matter volume (NWMV) demonstrate differing relationships with cognitive impairment in MS. Specifically, that NGMV tends to show strong associations that persist overtime [9] whilst NWMV shows less clear relationships [10]. Beyond this, studies investigating region-specific structural measures, including deep grey matter structures such as the thalamus, have further highlighted the potential relevance of anatomically specific measures for MS-related cognitive impairment [11]. This includes measures such as the corpus callosum index (CCI), a measure of corpus callosum morphology. The corpus callosum is a large white matter tract linking the hemispheres, with interhemispheric disconnection due to MS pathology being implicated as a contributor to cognitive decline in MS [12, 13]. This is in line with preliminary evidence which shows that CCI is strongly associated with cognitive impairment [14].

Recent studies in broader clinical contexts show that clinicians adjust diagnostic judgments regarding the presence or absence of cognitive impairment in patients depending on whether normative volumetric references flag low global or regional volumes, indicating that such markers are already influencing clinical decision-making regarding patients’ cognitive status [15]. Their clinical utility in MS, however, remains unclear, as a paucity of studies has evaluated the diagnostic accuracy of these neuroimaging markers for identifying cognitive impairment in this population. Therefore, there is a need to investigate the diagnostic performance of NBV, NGMV, NWMV, and CCI in distinguishing between cognitively impaired and non-impaired MS patients and to develop specific optimal brain volume cut-offs to identify those who are at risk of cognitive impairment.

Furthermore, the clinical translatability of neuroimaging markers NBV, NGMV, NWMV, and CCI remains limited because current findings have not been validated using routinely acquired clinical MRI datasets. As such, proposed MRI biomarkers have yet to achieve clinical translation, owing to the lack of validation in real-world cohorts and clinically relevant volumetric software such as Morphobox [16, 17]. If validated, routinely collected MRI data could provide a time- and cost-effective means of screening for cognitive impairment, addressing the current lack of reliable triage tools for referrals that currently constrain neuropsychological care and access.

This study investigated whether routinely collected structural neuroimaging markers can identify cognitive impairment in MS. We first examined the relationship between NBV, NGMV, NWMV, and CCI and cognition in an observational dataset of patients with MS, as assessed using the Brief Cognitive Assessment for Multiple Sclerosis (BICAMS), the standard cognitive battery used in MS research [18]. Using this same measure of cognitive impairment, we then examined how well these neuroimaging markers performed as screening tests for cognitive impairment. Finally, we exploratorily examined whether these neuroimaging markers might be associated with scores on additional cognitive tests not routinely used in MS research. We expected that, consistent with prior work, NBV, NGMV, and CCI would perform well as screening measures, while NWMV would not.

## Methods

### Study population

**Fig. 1:**
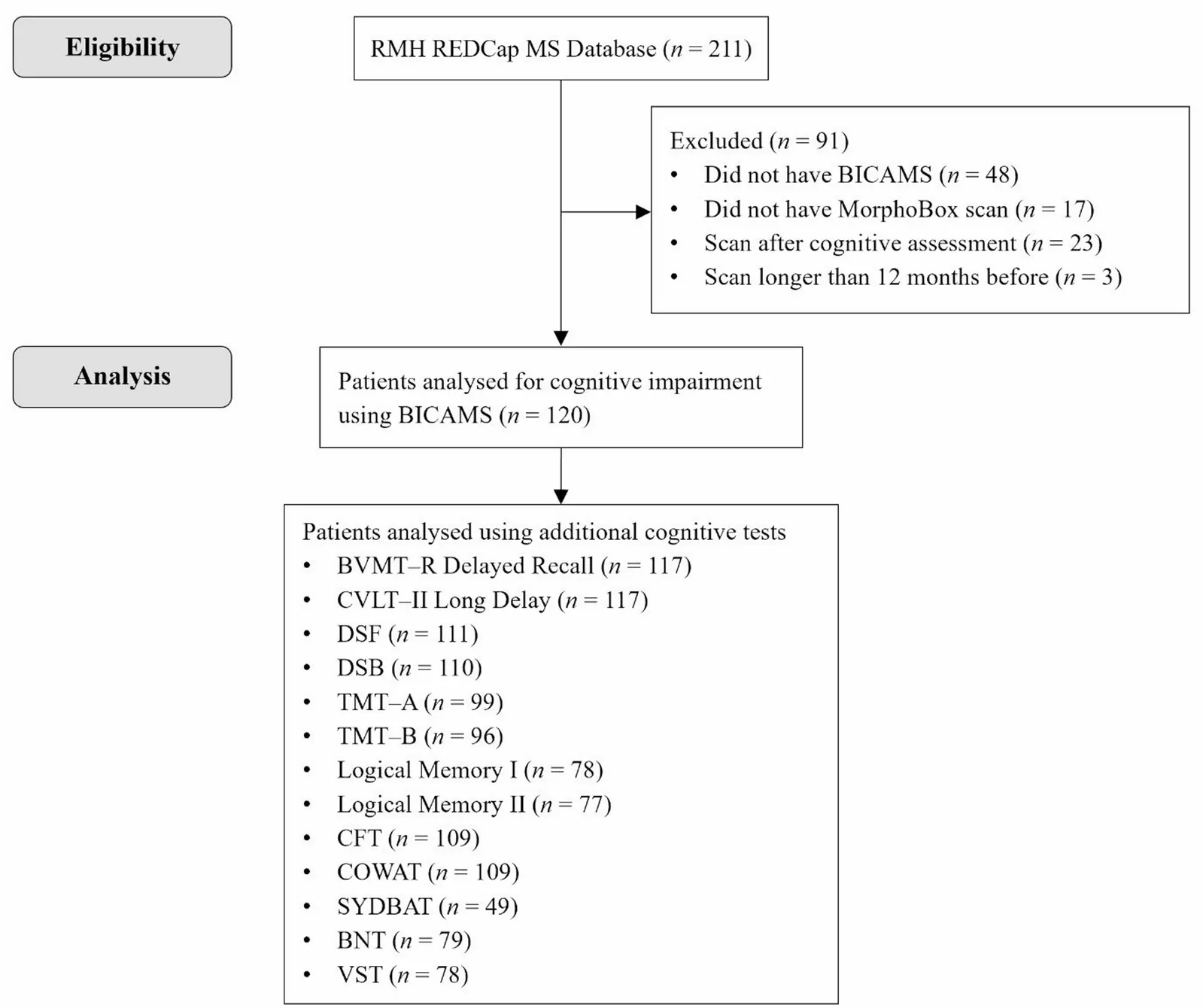
Flowchart of patient inclusion and analysis. Note: Patients with scans following cognitive assessment were excluded to avoid temporal ambiguity bias. For the additional cognitive tests, analyses used all available data on each measure, and the reported sample sizes therefore reflect the number of participants with usable scores that specific test. RMH = Royal Melbourne Hospital; REDCap = Research Electronic Data Capture; MS = multiple sclerosis; BICAMS = Brief International Cognitive Assessment for Multiple Sclerosis; BVMT–R = Brief Visuospatial Memory Test–Revised; CVLT–II = California Verbal Learning Test–second edition; DSF = Digit Span Forwards; DSB = Digit Span Backwards; TMT–A = Trail Making Test part A; TMT–B = Trail Making Test part B; CFT = Categorical Fluency Test; COWAT = Controlled Oral Word Association Test; SYDBAT = Sydney Language Battery; BNT = Boston naming test; VST = Victoria Stroop Test

This was a retrospective, observational, cohort study using clinical data collected from patients seen at the Cognitive Neuroimmunology Clinic at the Royal Melbourne Hospital (RMH) Neuroimmunology Centre, Melbourne, Australia, for patients seen between March 2020 and October 2024. Cognitive assessment scores and clinicodemographic information were recorded by the attending neuropsychologist, and imaging data were stored in a separate database by the attending radiologist. Data were screened for inclusion as outlined in Fig. 1. This study included patients with all MS types, diagnosed as clinically isolated syndrome (CIS), relapsing-remitting MS (RRMS), secondary progressive MS (SPMS), or primary progressive MS (PPMS) according to the 2017 revised McDonald criteria [19].

### Standard protocol approvals, registrations, and patient consents

The study was approved by the RMH Human Research Ethics Committee, which waived the need for patient consent for the collection, analysis, and publication of the retrospectively obtained and anonymised data for this non-interventional study (QA2021089). All research was conducted in accordance with the ethical standards of the 2024 revision of the Declaration of Helsinki [20].

### Measures

#### Primary psychometric cognitive measures

Cognitive impairment was assessed with the BICAMS [18]. Impairment was defined as performance at or below 1.5 *SD* on at least one BICAMS subtest [21].

The BICAMS battery included the Brief Visuospatial Memory Test–Revised (BVMT–R), total recall to evaluate immediate visual recall memory [22]. Patients were presented with a 2 × 3 array of abstract geometric figures. After each of the three 10-second learning trials, the array was removed, and patients were required to draw as many figures as possible in their correct locations. Raw scores (the number of correctly recalled designs across the three learning trials) were recorded and converted to *z* scores based on age-matched normative data [23]. The BVMT–R demonstrates high test–retest reliability in MS patients (*r* = .91) and high sensitivity (mean *d* = 1.03) [24].

The BICAMS battery included the California Verbal Learning Test–second edition (CVLT–II), total trials 1–5 to evaluate immediate verbal recall [25]. Patients were presented with a 16-item word list, with each item belonging to one of four semantic categories (four items per category). The list, randomly arranged but fixed in order, was read aloud to the patient five times. After each reading, patients were required to freely recall as many items as possible. Raw scores (the total number of words correctly recalled across the five learning trials) were recorded and converted to *z* scores based on normative data matched for age and sex. The CVLT–II demonstrates acceptable test–retest reliability among MS patients (*r* = .78) and high sensitivity (mean *d* = 0.89) [24].

The BICAMS battery included the Symbol Digit Modalities Test (SDMT) to evaluate information processing speed [26]. The oral version was administered. Patients were presented with a standard form displaying nine symbol–digit pairs at the top, followed by rows of randomised symbols. They were required to read aloud the digit corresponding to each symbol as quickly and accurately as possible within 90 s. The number of correct responses was recorded and converted to *z* scores using normative data matched for age and years of education [27]. The SDMT demonstrates high test–retest reliability in MS patients (*r* = .90) and high sensitivity (mean *d* = 1.11) [24].

#### Additional psychometric cognitive measures

Additional psychometric tests were included for exploratory analyses to comprehensively capture cognitive impairment. These included: BVMT–R delayed recall; CVLT–II long delay free recall; Digit Span Forwards (DSF) and Backwards (DSB); Trail Making Test (TMT), part A and B; Logical Memory I and II; Category Fluency Test (CFT); Controlled Oral Word Association Test (COWAT); Sydney Language Battery – naming subtest; Boston Naming Test (BNT); and Victoria Stroop Test (VST). Citations and detailed descriptions of administration, scoring, norming, and psychometric properties are provided in Appendix A of the Supplementary Materials.

#### Clinicodemographic information

Clinicodemographic variables were extracted from the electronic medical records and research databases. These included sex (male, female), MS subtype (or CIS), age (in years), education (in years), disease-modifying therapy (DMT), disease duration (in years), and the interval between MRI scan and cognitive testing (scan-to-test interval, in days).

The Expanded Disability Status Scale (EDSS) was included as a measure of disability severity widely used in clinical practice and research [28]. The scale evaluated physical and functional abilities, with a particular focus on mobility, and provided a score ranging from 0 (no disability) to 10 (death due to MS) in half-point increments. The patient was evaluated on eight functional systems: Pyramidal, cerebellar, brainstem, sensory, bowel and bladder, visual, cerebral, and other (each scale ranging from 0 to 5 or 6). The EDSS demonstrates good interrater reliability (κ = 0.32–0.76; agreement 71–87%) and intrarater reliability (κ ≈ 0.70) [29].

### Imaging

Scans were acquired at a single site using a 3-Tesla MRI scanner (Skyra; Siemens, Erlangen, Germany). The MRI protocol for brain demyelination included 3D T1-weighted MPRAGE imaging with voxel size of 1 mm isotropic; repetition time, 2,300 ms; echo time, 2.98 ms, inversion time, 900 ms; flip angle, 9°; and acquisition time, 5 min 9 s. Morphometric measures were obtained using the MorphoBox volumetric software (Syngo.via VB30B.36) [17]. The structural neuroimaging markers generated by MorphoBox were expressed as percentages relative to intracranial volume. No changes to the MRI scanner or imaging acquisition protocol occurred during the study inclusion period. De-identified MorphoBox outputs were manually transcribed, and data were filtered to include NBV, NGMV, NWMV, and CCI. Age-adjusted 10th-percentile cut-off values derived from the MorphoBox normative database were used to identify patients with below-normal brain volumes for each neuroimaging marker. To our knowledge, evidence specifically evaluating the generalisability of these methods across different imaging environments is currently limited.

### Statistical analysis

All data processing and statistical analysis were performed using R statistical computing software (Version 4.3.3) [30]. Descriptive statistics for clinicodemographic and neuroimaging characteristics across the sample and cognitive impairment groups were computed. Clinicodemographic variables were not subjected to formal inferential comparison as the study was not specifically powered to evaluate differences in these characteristics between groups.

General linear models (GLM) were used to compare mean group differences in neuroimaging measures between impaired and non-impaired patients. GLMs were also used to assess the associations between neuroimaging measures and BICAMS domain scores, as well as the additional psychometric test scores. Models included age and education as continuous confounder variables. Unstandardised and standardised regression coefficients with their bias-corrected and accelerated (BCa) bootstrapped 95% confidence intervals (CI) using 50,000 iterations. They were considered significant if the 95% CI did not include 0. For the analysis involving the additional psychometric tests, CIs were corrected for multiplicity using the Benjamini–Yekutieli false coverage rate procedure for the 13 exploratory models [31].

ROC analysis was conducted to evaluate the diagnostic performance of the neuroimaging measures in classifying cognitive impairment. Raw models (with neuroimaging marker only) and adjusted models (age, education, and neuroimaging marker) were tested. Optimal cut-off values were determined using Youden’s index. The area under the curve (AUC) with percentile bootstrapped 95% CIs (50,000 iterations) was deemed significant if the lower bound of the 95% CI exceeded 0.50. Satisfactory performance was defined as AUC ≥ 0.70 [32].

The diagnostic performance of the neuroimaging measures in classifying cognitive impairment was also evaluated using the percentile-based volumetric cut-offs derived from MorphoBox’s age-matched normative references [17]. For each marker, patients were dichotomised according to whether their volume fell below the age-adjusted 10th percentile threshold. Odds ratios (OR) with 95% CIs were estimated to quantify the correlation between below-threshold status and impairment. BCa bootstrapped ORs (50,000 iterations) were deemed significant if the 95% CI excluded 1. Complete details of the statistical analyses are described in Appendix B of the Supplementary Materials.

## Results

### Sample characteristics

Table 1 presents the clinicodemographic and imaging characteristics of the sample according to cognitive impairment status. Cognitive impairment was detected in 35% of the sample, with impairment in BVMT–R for 24 patients (20%), CVLT–II for 14 (12%), and SDMT for 25 (21%). 

**Table 1:** Mean and standard deviations of clinicodemographic and neuroimaging characteristics according to cognitive impairment status

Variable	Overall (*N* = 120)	Impaired (*n* = 42)	Non-Impaired (*n* = 78)
Clinicodemographic			
Sex, *n*	93 F, 27 M	33 F, 9 M	60 F, 18 M
MS Type, *n*	114 RR, 1 SP, 5 PP	40 RR, 2 PP	74 RR, 1 SP, 3 PP
Age (years)	42.48 (12.45)	39.21 (13.08)	44.24 (11.82)
Education (years)	13.43 (2.51)	12.69 (2.41)	13.83 (2.48)
EDSS, median (MAD)	2.00 (1.48)	2.25 (1.11)	2.00 (1.48)
Disease Duration (years)	10.03 (8.70)	10.82 (8.49)	9.6 (8.83)
Scan-to-Test Interval (days)	128.99 (87.91)	127.29 (76.41)	129.91 (93.98)
Imaging			
NBV (%)	77.46 (3.75)	76.72 (3.98)	77.86 (3.59)
NGMV (%)	47.4 (2.77)	47.67 (2.91)	47.26 (2.71)
NWMV (%)	30.05 (2.46)	29.05 (2.37)	30.59 (2.36)
CCI (%)	3.72 (0.56)	3.59 (0.59)	3.79 (0.53)
DMT			
Ocrelizumab, *n* (%)	38 (32)	11 (26)	17 (35)
Natalizumab, *n* (%)	29 (24)	13 (31)	16 (21)
Cladribine, *n* (%)	20 (17)	8 (19)	12 (16)
Fingolimod, *n* (%)	10 (8)	2 (5)	8 (10)
Dimethyl Fumarate, *n* (%)	4 (3)	1 (2)	3 (4)
Ofatumumab, *n* (%)	2 (2)	1 (2)	1 (1)
Teriflunomide, *n* (%)	2 (2)	0 (0)	2 (3)
Alemtuzumab, *n* (%)	1 (1)	0 (0)	1 (1)
Glatiramer Acetate, *n* (%)	1 (1)	0 (0)	1 (1)
None, *n* (%)	12 (10)	6 (14)	6 (8)

Descriptive statistics are *M* (*SD*) unless otherwise specified

*F* Female, *M* Male, *MS* Multiple sclerosis, *RR* Relapsing remitting, *SP* Secondary progressive, *PP* Primary progressive, *EDSS* Expanded disability status scale, *MAD* Median absolute deviation, *NBV* Normalised brain volume, *NGMV* Normalised grey matter volume, *NWMV* Normalised white matter volume, *CCI* Corpus callosum index, *DMT* Disease-modifying therapy

### Group differences in neuroimaging markers by cognitive impairment status

Group differences in neuroimaging measures between impaired and non-impaired patients were examined using two-sample comparisons of means via GLMs. As shown in Table 2, NBV and NWMV were lower in the impaired patients after adjusting for age and education. There was no evidence for mean differences in NGMV or CCI. Full details of these analyses are reported in Appendix C of the Supplementary Materials.

**Table 2:** Two-sample comparisons of means via GLM of neuroimaging markers according to cognitive impairment status

Neuroimaging Marker	*B* [95% CI]	β [95% CI]	*R* ^2^_adj_	ΔR^2^
NBV	–2.06* [–3.35, − 0.81]	–0.55* [–0.86, − 0.23]	.28	.06
NGMV	–0.41 [–1.17, 0.38]	–0.15 [–0.42, 0.14]	.49	.00
NWMV	–1.65* [–2.60, − 0.77]	–0.67* [–0.99, − 0.33]	.07	.09
CCI	–0.22 [–0.43, 0.01]	–0.40 [–0.78, 0.00]	.03	.03

Age and education were included in all models. All *R*^2^_adj_ and Δ*R*^2^ values are from unstandardised models. GLM = generalised linear model

*B* = unstandardised coefficient, *CI* Confidence interval, *β* Standardised coefficient, *NBV* Normalised brain volume, *NGMV* Normalised grey matter volume, *NWMV* Normalised white matter volume, *CCI* Corpus callosum index, non-impaired = 0, impaired = 1

*indicates 95% CI does not include 0

**Fig. 2:**
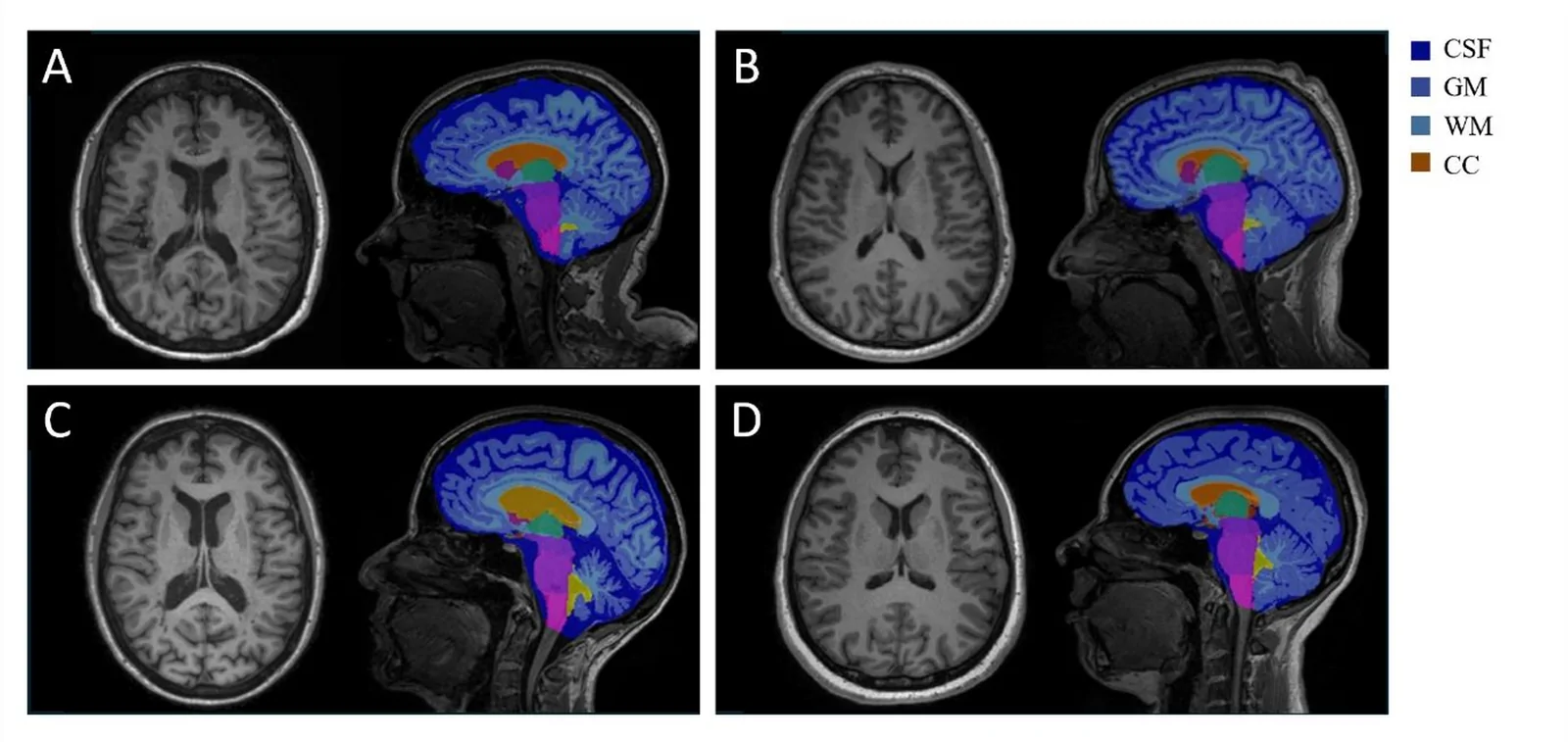
Example MorphoBox outputs and segmentations according cognitive impairment status and NBV values. Note: Each panel displays MorphoBox outputs from a different patient, with the axial slice on the left, and the sagittal slice on the right. Coloured segmentation overlays represent tissue classifications generated by MorphoBox; tissue labels are defined in the legend for interpretability. NBV = normalised brain volume. CSF = cerebrospinal fluid; GM = grey matter; WM = white matter; CC = corpus callosum. Panel **A**: Cognitively non-impaired patient with the lowest NBV in the sample. Panel **B**: Cognitively non-impaired patient with the highest NBV in the sample. Panel **C**: Cognitively impaired patient with the lowest NBV in the sample. Panel **D**: Cognitively impaired patient with the highest NBV in the sample

To illustrate the absence of strong relationships between cognitive status and NBV, Fig. 2 displays MorphoBox segmentations from four representative patients.

### Association between neuroimaging markers and cognitive test scores

The relationship between neuroimaging markers and BICAMS subtest scores was investigated using GLMs. As shown in Table 3, NBV, NWMV, and CCI emerged as significant predictors after adjusting for age and education, although the pattern of association varied across specific subtests. Full details are reported in Appendix D of the Supplementary Materials.

**Table 3:** GLMs examining neuroimaging markers as predictors of cognitive subtest scores

Model	*B* [95% CI]	β [95% CI]	*R* ^2^_adj_	Δ*R*^2^	AIC
BVMT–R					
NBV	0.65* [0.26, 1.06]	0.33* [0.13, 0.51]	.22	.08	316.07
NGMV	0.47 [–0.11, 1.17]	0.18 [–0.05, 0.42]	.15	.02	326.45
NWMV	0.85* [0.33, 1.33]	0.28* [0.11, 0.43]	.22	.08	316.73
CCI	1.77 [–0.38, 3.88]	0.14 [–0.03, 0.28]	.15	.02	326.30
CVLT–II					
NBV	0.57 [–0.01, 1.15]	0.18 [–0.01, 0.35]	.18	.02	322.60
NGMV	0.39 [–0.49, 1.22]	0.09 [–0.11, 0.29]	.16	.00	325.65
NWMV	0.76* [0.01, 1.53]	0.16 [0.00, 0.30]	.18	.03	322.53
CCI	1.01 [–2.42, 4.27]	0.05 [–0.12, 0.19]	.16	.00	325.93
SDMT					
NBV	1.37* [0.77, 1.88]	0.43* [0.24, 0.57]	.32	.14	300.67
NGMV	0.88 [–0.14, 1.76]	0.21 [–0.03, 0.41]	.19	.02	321.08
NWMV	1.87* [1.09, 2.63]	0.39* [0.23, 0.52]	.33	.15	299.15
CCI	4.20* [0.92, 7.53]	0.20* [0.04, 0.34]	.21	.04	318.44

Age and education were included in all models. All *R*^2^_adj_, Δ*R*^2^, and AIC values are from unstandardised models

*GLM* General linear model, *B* = unstandardised coefficient, *CI* Confidence interval, *β* Standardised coefficient, *AIC* Akaike information criterion, *BVMT–R* Brief Visuospatial Memory Test–Revised, *CVLT–II* California Verbal Learning Test–second edition, *SDMT* Symbol Digits Modalities Test, *NBV* Normalised brain volume, *NGMV* Normalised grey matter volume, *NWMV* Normalised white matter volume, *CCI* Corpus callosum index

*indicates 95% CI does not include 0

### Accuracy of neuroimaging markers at classifying cognitive impairment status

**Fig. 3:**
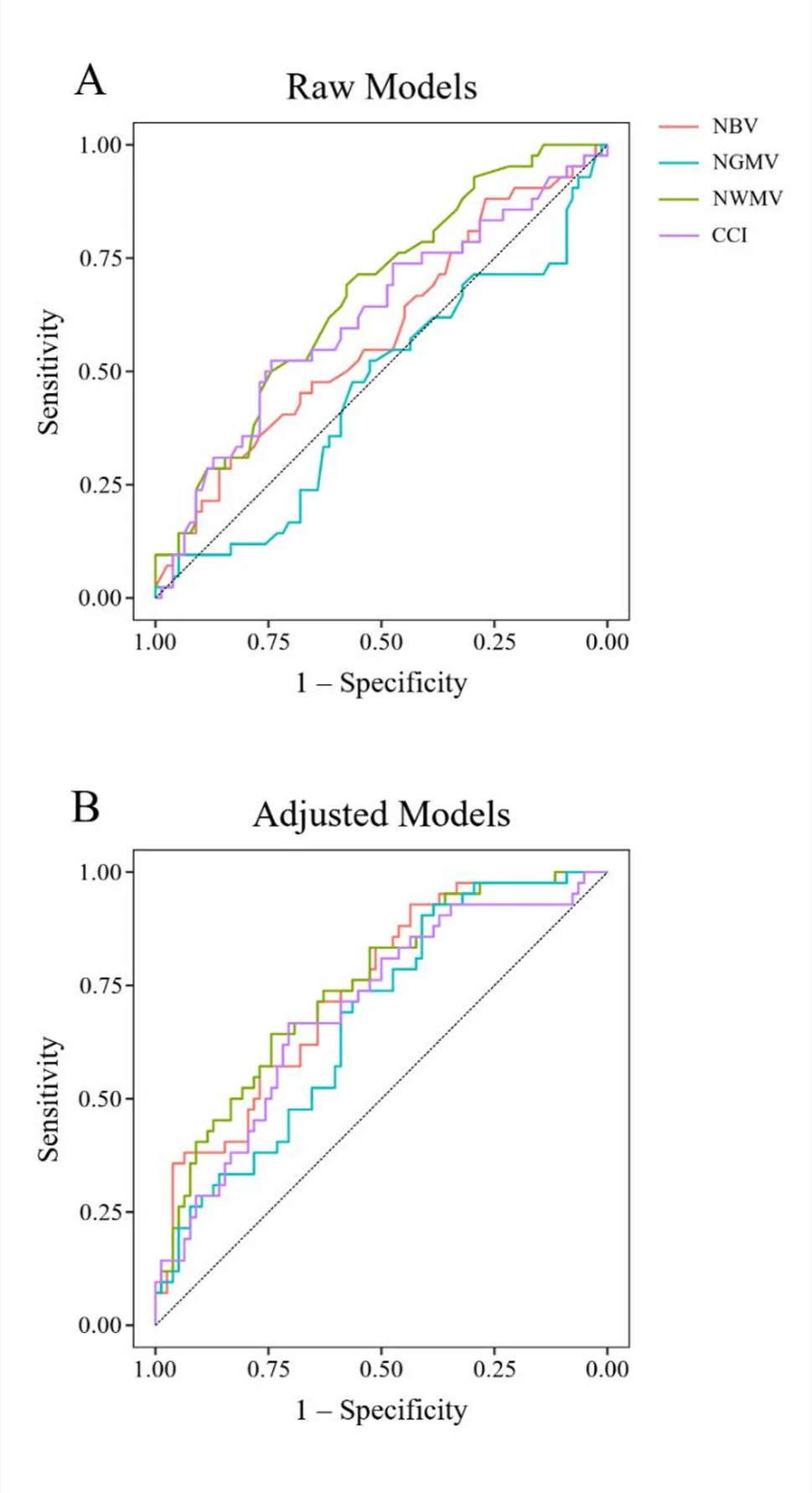
ROC curves for neuroimaging markers classifying cognitive impairment. Note: ROC = receiver operating characteristic; NBV = normalised brain volume; NGMV = normalised grey matter volume; NWMV = normalised white matter volume; CCI = corpus callosum index. Panel **A**: Raw models which show values of the neuroimaging measures without adjusting for age and education. Panel **B**: Adjusted models which include the effect of age and education

The general screening performance of NBV, NGMV, NWMV, and CCI in classifying cognitive impairment was assessed using ROC analysis. As shown in Table 4, only raw NWMV and CCI demonstrated significant discriminative ability. However, the sensitivity and specificity metrics were poor. The screening performance of models adjusted to also include demographic information known to confound the effects of both imaging and cognitive performance measures were also assessed [33]. These adjusted analyses reflect multivariate models combining neuroimaging measures with age and education, rather than the performance of neuroimaging markers in isolation. For the adjusted models, all imaging measures showed significant discriminative ability. Only adjusted NBV and NWMV performed well as screening measures, with sensitivities ≥ 0.90. The screening performances of these metrics are shown in Fig. 3.

**Table 4:** ROC analysis showing cut-offs and diagnostic accuracy of neuroimaging measures for identifying cognitive impairment status

Model	AUC [95% CI]	Cut-Off	Sensitivity	Specificity	PPV	NPV
Raw						
NBV	.58 [0.47, 0.69]	< 80.65%	.88	.27	.39	.81
NGMV	.46 [0.35, 0.57]	< 47.35%	.52	.53	.37	.67
NWMV	.67* [0.57, 0.76]	< 30.30%	.69	.58	.47	.78
CCI	.62* [0.51, 0.72]	< 3.54%	.52	.74	.52	.74
Adjusted						
NBV	.74* [0.65, 0.82]	< 80.18%	.93	.44	.47	.92
NGMV	.67* [0.58, 0.77]	< 50.46%	.90	.41	.45	.89
NWMV	.75* [0.65, 0.83]	< 29.67%	.64	.74	.57	.79
CCI	.70* [0.60, 0.79]	< 3.45%	.67	.71	.55	.80

Raw models show values of the neuroimaging measures without adjusting for age and education. Adjusted models include the effect of age and education

*ROC* Receiver operating characteristic, *AUC* Area under the curve, *CI* Confidence interval, *PPV* Positive predictive value, *NPV* Negative predictive value, *NBV* Normalised brain volume, *NGMV* Normalised grey matter volume, *NWMV* Normalised white matter volume, *CCI* Corpus callosum index, non-impaired = 0, impaired = 1; not below cut-off = 0, below cut-off = 1

*indicates lower bound of 95% CI > 0.50

The diagnostic performance of NBV, NGMV, NWMV, and CCI in classifying cognitive impairment using the percentile-based volumetric cut-offs derived from MorphoBox age-matched normative references are shown in Table 5. Only lower volumes for NBV and NWMV were associated with cognitive impairment, although the sensitivities were low (< 0.30).

**Table 5:** Diagnostic accuracy of neuroimaging markers using MorphoBox age-matched 10th percentile normative cut-offs to classify cognitive impairment

Neuroimaging Marker	OR [95% CI]	Sensitivity	Specificity	PPV	NPV
NBV	5.60* [1.50, 20.08]	.26	.94	.69	.70
NGMV	1.97 [0.30, 11.84]	.10	.95	.50	.66
NWMV	3.16* [1.09, 8.75]	.29	.88	.57	.70
CCI	2.36 [0.95, 5.63]	.36	.81	.50	.70

Observed cell counts fell below 20 for all markers, indicating that diagnostic accuracy metrics may be unstable

*CI* Confidence interval, *PPV* Positive predictive value, *NPV* Negative predictive value, *NBV* Normalised brain volume, *NGMV* Normalised grey matter volume, *NWMV* Normalised white matter volume, *CCI* Corpus callosum index, non-impaired = 0, impaired = 1; not below cut-off (≥ 10th percentile) = 0, below cut-off (< 10th percentile) = 1

*indicates 95% CI does not include 1

### Exploratory analysis of additional cognitive tests

The relationship between imaging markers and additional cognitive tests were investigated using GLMs adjusted for age and education. NBV significantly predicted BVMT–R Delayed Recall (*b* = 0.22, 95% CI [0.04, 0.43], *R*^2^_adj_ = 0.20), TMT–A (*b* = − 0.80, 95% CI [–1.58, − 0.02], *R*^2^_adj_ = 0.15), LM II (*b* = 0.59, 95% CI [0.04, 1.08], *R*^2^_adj_ = 0.05), and CFT (*b* = 0.40, 95% CI [0.06, 0.69], *R*^2^_adj_ = 0.00). NGMV significantly predicted TMT–B (*b* = − 3.75, 95% CI [–6.98, − 0.16], *R*^2^_adj_ = 0.03). NWMV significantly predicted BVMT–R Delayed Recall (*b* = 0.30, 95% CI [0.07, 0.53], *R*^2^_adj_ = 0.13), and CFT (*b* = 0.49, 95% CI [0.05, 0.88], *R*^2^_adj_ = 0.05). Full details are reported in Appendix E of the Supplementary Materials.

## Discussion

Cognitive impairment is common in MS, yet neuropsychological care and access are currently constrained by the lack of reliable triage tools for referrals [1]. Based on reported associations between NBV, NGMV, NWMV, and CCI with cognitive impairment in MS, this study investigated whether routinely collected structural neuroimaging markers could be used to identify MS patients at elevated risk of cognitive impairment [5]. We found that impaired patients had lower NBV and NWMV, and that NBV, NWMV, and CCI were associated with scores on at least one BICAMS subtest. While raw CCI and NWMV volumes showed statistically significant classification of cognitive impairment, screening metrics did not meet acceptable thresholds for clinical screening. When combined with other clinical information however, all metrics showed stronger and statistically significant relationships with cognitive impairment. The implications of the findings from these adjusted models, including the rationale, interpretation, and clinical relevance are discussed in the Limitations and Future Directions section below. Using the age-adjusted normative cut-off scores from MorphoBox, however, did not show this same improvement in screening performance.

### Correlational findings between neuroimaging markers and cognitive impairment

This study found that previously reported correlations between NBV, NGMV, NWMV, and CCI and cognition in MS did not replicate when tested in a representative clinical sample with adjustment for demographic confounders. Moderate associations were observed between NBV and CCI with some measures of cognitive performance, which is generally consistent with previous studies [7, 14]. However, unlike many previous findings, NGMV showed no clear relationship with cognition, while NWMV demonstrated the most consistent associations with cognitive measures, with effects ranging from moderate to large magnitude [9, 10]. These findings are somewhat unexpected given the substantial literature supporting associations between grey matter structures, such as the thalamus and hippocampus, and cognitive impairment in MS [11, 34]. Differences between the current findings and previous literature may reflect methodological differences, including the use of a representative clinical population and MorphoBox-derived neuroimaging metrics, which have not been employed in many of the previous studies.

### Diagnostic accuracy of neuroimaging markers at identifying cognitive impairment in MS

This study builds upon previous findings by suggesting that neuroimaging markers commonly proposed as indicators of cognitive impairment in MS may not accurately distinguish cognitively impaired from non-impaired patients well enough to be used for clinical cognitive screening purposes. Despite routinely collected raw markers such as NBV and CCI yielding statistically significant AUC values, their diagnostic performance indices were modest at best, with no marker meeting conventional thresholds for clinically acceptable screening accuracy. Importantly, these diagnostic metrics should be interpreted cautiously as they likely represent optimistic estimates of accuracy given that they were derived and tested in the same sample and may therefore reflect some degree of overfitting. Validation in an independent cohort would almost certainly yield weaker results [35]. These findings indicate that correlational associations reported in the literature may not directly translate into clinically robust diagnostic tools for identifying cognitive impairment at the individual patient level. Despite the lack of studies assessing the isolated diagnostic performance of routinely collected neuroimaging markers, clinicians have been shown to rely on correlational findings to make diagnostic judgements regarding the presence or absence of cognitive impairment in clinical practice depending on whether normative volumetric references flag low global or regional volumes [15]. The present results shed light on the possible limitations of this practice, suggesting that it may assume that group-level correlations reported in the literature apply to clinical cohorts and hold at the individual level, an assumption the contingency analysis showed was not consistently supported and may be unstable, respectively.

### Exploratory findings

The exploratory analyses suggested that routinely collected neuroimaging markers may show limited associations with cognitive domains beyond those assessed by the BICAMS. NGMV and CCI were weakly correlated with the additional cognitive test scores, whereas NBV and NWMV showed modest associations. However, these correlations appeared to be mostly confined to domains overlapping with those that they correlated with in the BICAMS, such as visual memory and information-processing speed. Although this pattern could imply that these neuroimaging markers are most sensitive to the cognitive domains encompassed by the BICAMS, such an interpretation remains speculative. Domain-level inferences were not directly examined in this study, as correlations were tested at the level of individual cognitive tests rather than aggregated cognitive domains defined by established frameworks, such as the International Classification of Cognitive Disorders in MS [21]. Nevertheless, these exploratory findings extend previous literature by showing that, within this clinical sample, routinely collected structural neuroimaging markers showed limited correlations with cognitive domains beyond those assessed by the BICAMS.

### Limitations and future directions

This study has several strengths that support the clinical translatability of its findings. These include the use of a representative clinical sample with a prevalence of cognitive impairment consistent with epidemiological estimates (~ 30%), strengthening the generalisability of the findings to clinical populations [2]. Additionally, the study utilised MorphoBox-derived metrics, an automated neuroimaging volumetry tool integrated into clinical workflows, which remains relatively underexplored in cognitive screening studies despite its widespread use in routine monitoring for MS [17].

However, this study is not without limitations. Firstly, the retrospective, single-centre design may have reduced sample representativeness, as the study relied on existing patients from a single specialist cognitive clinic. Although recruitment from a specialised cognitive service may be expected to overrepresent cognitively impaired patients, the prevalence observed in the current cohort (~ 30%) was comparable to epidemiological estimates in the general MS population [2]. However, potential selection bias may still be present as patient inclusion was dependant on the availability of complete data.

Furthermore, the sample size represented a limitation for the diagnostic accuracy analyses. Specifically, the relatively modest sample size may have reduced the precision and stability of diagnostic accuracy estimates and derived cut-off values, consistent with the instability noted in Table 5 for the contingency analysis [36, 37]. Future studies should seek to validate these findings in larger, independent cohorts to address these concerns. Such efforts would also address the lack of external validation of ROC-derived cut-offs.

Additionally, the relationships between neuroimaging measures and cognitive performance were examined cross-sectionally, limiting the ability to determine whether these findings remain consistent over time. Longitudinal studies would provide stronger evidence by establishing temporal relationships and allowing the prognostic utility of these markers to be evaluated, helping determine whether they predict subsequent cognitive decline [38].

This study defined cognitive impairment using the widely used threshold suggested by Hancock et al. [21]. However, it is important to acknowledge that this relatively liberal threshold may have increased estimated prevalence within the sample and influenced classification performance [39]. Inclusion of patients with borderline cognitive deficits in the impairment group may have increased heterogeneity and reduced separation between impaired and non-impaired patients, potentially affecting sensitivity, specificity and overall discrimination [4].

This study also did not investigate group interactions when examining associations between neuroimaging markers and cognitive performance. It is possible that the absence of observed associations for markers such as NGMV could reflect heterogeneity in the relationship between NGMV and cognitive performance across cognitive impairment groups, whereby differing associations between groups may have been attenuated by analyses fitting a single average slope across the overall cohort [40]. Future studies incorporating interaction effects may help strengthen the interpretation of the current study findings by determining whether relationships between neuroimaging markers and cognitive performance differ between cognitively impaired and non-impaired MS patients.

A final key constraint was the absence of high-quality normative data that fully standardise BICAMS scores for both age and education. Although the best available normative data were used to standardise cognitive scores, they did not fully adjust for both age and education, which limited the ability to both assess and apply routinely collected neuroimaging markers for their independent diagnostic performance [22, 25, 26]. This study aimed to overcome these issues by means of statistical adjustment by including age and education in ROC models to observe how the relationships between neuroimaging and cognitive markers were expressed while accounting for their effects. However, because such adjustment in the ROC classification models simply reflects multivariate models combining the effects of neuroimaging measures with age and education, it obscures the isolated contribution of neuroimaging markers in classifying cognitive impairment. Therefore, it remains unclear how raw neuroimaging measures perform in this context [41]. Importantly, regression-derived formulas would need to be applied for these cut-offs to be used as a screening tool for cognitive impairment in clinical practice. This requirement fundamentally undermines clinical practicality, as the strength of a screening tool lies in its ease of use. Future research should therefore prioritise developing such comprehensive normative data to better assess the diagnostic value of routinely collected neuroimaging markers and clarify their potential as a clinically useful screening tool for cognitive impairment in MS.

## Conclusion

Cognitive impairment remains a disabling yet under-addressed symptom of MS, and efficient detection is crucial for ensuring timely and equitable access to neuropsychological care [1]. By evaluating routinely collected MRI-derived neuroimaging markers, this study provides preliminary evidence regarding their potential limitations as tools for cognitive screening in real-world clinical settings. Despite their strong theoretical rationale, the present study could not find strong evidence that routinely collected neuroimaging markers could perform sufficiently as a practical solution for cognitive screening in MS, and that further investigation is warranted to comprehensively understand their utility as surrogates for cognitive impairment in MS. Nevertheless, this work contributes to understanding the potential limits of using routinely collected structural neuroimaging markers to identify cognitive impairment in MS, and highlights the methodological considerations that should guide future efforts to develop clinically viable screening approaches.

## Supplementary Information

Supplementary Material 1

## Data Availability

De-identified data and complete analysis may be accessed from the following online data depository: 10.17605/OSF.IO/5BNG6.
