# Supplementary material for: The use of routinely collected structural neuroimaging to identify cognitive impairment in multiple sclerosis
Source: BMC Neurol. 2026 Jun 17;26:544. doi: 10.1186/s12883-026-05085-z (PMC13520496; doi:10.1186/s12883-026-05085-z)
Supplement: Supplementary file 1 [file 12883_2026_5085_MOESM1_ESM.docx]

**Appendix A**

**Detailed Description of Additional Cognitive Tests**

***BVMT–R, Delayed Recall***

The delayed recall trial of the BVMT–R is described as a measure of delayed visual memory [1]. After a 25-minute delay following the learning trials and immediate visual recall testing described under the BVMT–R, patients were asked again to reproduce the figures from memory. The number of figures correctly in the accurate location was recorded and converted to a *z* score based on age-matched normative data [2]. This test demonstrates high test–retest reliability (*r =* .81) and interrater reliability (*r* = .93).

***CVLT–II, Long Delay***

The long-delay free recall trial of the CVLT–II is described as a measure of delayed verbal memory [3]. After a 20-minute delay following the learning trials and immediate verbal recall testing, patients were asked to recall the original word list. The number of correctly recalled words from the original list was recorded and transformed into a *z* score based on normative data matched for age and sex. This test demonstrates high test–retest reliability (*r* = .80) and internal consistency (α = .94).

***Wechsler Adult Intelligence Scale–Fourth Edition, Digit Span Forwards***

The Digit Span Forwards (DSF) subtest of the Wechsler Adult Intelligence Scale–fourth edition (WAIS–IV) is described as a measure of attention, immediate verbal recall, and auditory processing [4]. Patients were read sequences of digits at a rate of one per second and instructed to repeat them in the same order. Sequence length increased progressively until the participant failed two consecutive sequences. The last correct sequence length was recorded and converted to a scaled score based on age-matched normative data. This subtest demonstrates good internal consistency (median α = .88) and test–retest reliability (*r* = .82).

***WAIS–IV, Digit Span Backwards***

The Digit Span Backwards (DSB) subtest of the WAIS–IV is described as a measure of working memory, mental manipulation, and executive functioning [4]. Patients listened to sequences of digits and were instructed to repeat them in reverse order. Sequence length increased with each correct response until the participant failed two consecutive sequences. The last correct sequence length was recorded and converted to a scaled score based on age-matched normative data. This subtest demonstrates good internal consistency (median α = .89) and test–retest reliability (*r* = 82).

***Trail Making Test***

The Trail Making Test (TMT) included parts A and B to measure processing speed and executive control, respectively [5]. In part A, patients were instructed to draw a line connecting 25 encircled numbers in ascending order as quickly as possible without lifting their pencil from the paper. In part B, patients were instructed to do the same but by alternating between numbers and letters in ascending sequence (e.g., 1-A-2-B, etc.). Time to completion was recorded for each part, with longer completion times indicating poorer performance. Raw scores for each part were separately converted to *z* scores based on normative data matched for age and years of education [6]. This test demonstrates good test–retest reliability (*r* = .79–.89).

***Wechsler Memory Scale–Fourth Edition, Logical Memory***

The Wechsler Memory Scale–Fourth Edition (WMS–IV), logical memory subtest is described as a measure of episodic verbal memory and includes components for immediate (logical memory I) and delayed recall (logical memory II) [7]. Patients were first read Story A aloud and asked to recall it immediately, followed by Story B, which was also recalled immediately (immediate recall). After a 20-minute delay, they were asked to recall the stories again without cues (delayed recall). The number of story details correctly recalled and recognised were recorded. Raw scores for each index were converted to scaled scores based on age-matched normative data. This subtest demonstrates good internal consistency (α = .79–88) and test–retest reliability (*r* = .72–.81).

***Category Fluency Test***

The Category Fluency Test (CFT) is described as a measure of semantic verbal fluency [8]. Patients were asked to generate as many words as possible within 60 seconds that belonged to a specified semantic category (e.g., animals). Patients were informed that proper nouns (e.g., places and names), repetitions, and morphological variants (e.g., fast, faster, fastest) were not counted as correct responses. The total number of correct responses was recorded and converted to *z* scores based on normative data matched for age and years of education [9]. This test demonstrates good construct validity and acceptable test–retest reliability (*r* ≈ .70–.80) [10,11].

***Controlled Oral Word Association Test***

The Controlled Oral Word Association Test (COWAT) is described as a measure of phonemic verbal fluency [8]. Patients were instructed to generate as many unique words as possible beginning with a specified letter (typically F, A, and S) within 60 seconds per letter. Patients were informed that proper nouns, repetitions, and morphological variants were not counted as correct responses. The number of total correct responses were recorded and converted to *z* scores based on normative data matched for age and years of education [9]. This test demonstrates good construct validity and acceptable test–retest reliability (*r* ≈ .70–.84) [12].

***Sydney Language Battery, Naming Test***

The Sydney Language Battery (SYDBAT) naming test is described as a measure of lexical retrieval [13]. Patients were presented with colour photographs of common objects one at a time and asked to name each aloud. The test includes 30 items, and each was presented individually without a time limit. If a participant failed to respond or gave an incorrect response, no cues were provided. The total number of correct responses was recorded and were converted to z-scores based on normative data matched for age and years of education. This test demonstrated high convergent validity (*r* = .95), and test–retest reliability (*r* = .89–.97).

***Boston Naming Test***

The Boston Naming Test (BNT) is described as a measure of confrontational naming ability [14]. Patients were shown a series of 60 black-and-white line drawings representing increasingly uncommon objects and asked to name each item aloud. If a participant failed to produce the correct name within 20 seconds, standardised semantic cues were provided. If the participant still could not provide the correct name, a phonemic cue was provided. Scoring was based on the number of correct spontaneous responses. Raw scores for each index were interpreted using normative data matched for age and years of education. This test demonstrates good internal consistency (α ≈ .80–.90) and high test–retest reliability (*r* = .77–.93) [10,11].

***Victoria Stroop Test, Colour Condition***

The colour condition of the Victoria Stroop Test (VST) is described as a measure of possessing speed and visual attention [15]. In this baseline condition, participants name the colour of dot stimuli as quickly as possible. Patients were required to respond as quickly as possible. Scoring was based on the time (in seconds) to complete each task. Raw scores for each index were converted to *z* scores based on normative data matched for age, sex, and years of education. This test demonstrates high test–retest reliability (*r* ≈ .79–.91) [11].

**Appendix B**

**Detailed Description of Statistical Analysis**

All data processing and statistical analysis were performed using R statistical computing software (Version 4.3.3) [16].

***Descriptive Statistics***

Frequencies, means, standard deviations, medians, and mean absolute deviations were computed for clinicodemographic variables including sex, MS type, education, EDSS, scan-to-test interval, disease duration, and neuroimaging measures.

***Group Differences in Neuroimaging Markers by Cognitive Impairment Status***

General linear models (GLM) were used to compare mean group differences in neuroimaging measures between impaired and non-impaired patients. Models included age and education as confounders. Model assumptions of linearity, homoscedasticity, normality of residuals, and multicollinearity were examined prior to analysis. Unstandardised and standardised regression coefficients with their 95% CIs, adjusted *R*^2^, Δ*R*^2^ (the increase in explained variance after the neuroimaging measure was added to the baseline model containing only age and education), and Akaike information criterion (AIC) values were reported. Bias-corrected and accelerated (BCa) bootstrapped regression estimates were obtained using 50,000 iterations to ensure robust inference. Estimates were considered significant if the 95% CI did not include 0.

***Association Between Neuroimaging Markers and Cognitive Test Scores***

GLMs were used to assess the predictive nature of neuroimaging measures for BICAMS domain scores. Inspection of standardised BICAMS domain score distributions revealed floor effects. To preserve meaningful variability, raw scores were used in analyses. Each neuroimaging measure was entered separately for each BICAMS domain. Model assumptions of linearity, homoscedasticity, normality of residuals, and multicollinearity were examined. Unstandardised and standardised regression coefficients with their 95% CIs, adjusted *R*^2^, Δ*R*^2^, and AIC values were reported. BCa bootstrapped regression parameters were obtained using 50,000 iterations, deemed significant if the 95% CI did not include 0.

***Accuracy of Neuroimaging Markers at Classifying Cognitive Impairment Status***

**Data-Driven Optimal Cut-Off Analysis.** ROC analysis was conducted to evaluate the general diagnostic performance of NBV, NGMV, NWMV, and CCI in classifying cognitive impairment. Raw models (with neuroimaging marker only) and adjusted models (age, education, and neuroimaging marker) were tested. Optimal cut-off values were determined using Youden’s index. Assumptions of ROC analysis were evaluated, including the representativeness of the sample’s rate of cognitive impairment relative to base rates of impairment. The area under the curve (AUC) with its 95% CIs, optimal cut-off values, sensitivity, specificity, positive predictive value (PPV), and negative predictive value (NPV) were reported. A percentile bootstrapping method with 50,000 iterations was used for the AUC, deemed significant if the lower bound of the AUC 95% CI exceeded .50. Thresholds were defined as AUC ≥ .70 indicating satisfactory performance [17].

**Predefined Normative Cut-Off Analysis.** The diagnostic performance of NBV, NGMV, NWMV, and CCI in classifying cognitive impairment was also evaluated using the percentile-based volumetric cut-offs derived from MorphoBox’s age-matched normative references [18]. For each marker, patients were dichotomised according to whether their volume fell below the age-adjusted 10th percentile threshold embedded in the MorphoBox volumetric software. Assumptions were evaluated, including observed cell counts being ≥ 20 within contingency tables to ensure the stability of diagnostic accuracy metrics [19]. Odds ratios (OR) with 95% CIs were estimated to quantify the correlation between below-threshold status and impairment. BCa bootstrapped ORs were obtained using 50,000 iterations, deemed significant if the 95% CI excluded 1. Diagnostic accuracy metrics (sensitivity, specificity, PPV, and NPV) were computed.

***Exploratory Analysis***

GLMs were used to assess the predictive value of neuroimaging measures for additional cognitive test scores. For consistency with the primary analyses, raw scores were used, as their distributions were comparable to standardised scores. Each neuroimaging measure was entered separately for each cognitive test score. Model assumptions were examined prior to analysis. Unstandardised and standardised regression coefficients with their 95% CIs, adjusted *R*^2^, Δ*R*^2^, and AIC values were presented. BCa bootstrapped linear regression parameters were obtained using 50,000 iterations, with CIs corrected for multiplicity using the Benjamini–Yekutieli false coverage rate procedure for the 13 exploratory models [20]. Estimates were treated as discoveries if their FCR-adjusted confidence interval did not include 0.

**Appendix C**

**Detailed Results for the Two-Sample Comparisons of Means**

**Table C.1**

*Detailed Results of the Two-Sample Comparisons of Means via GLM of Neuroimaging Markers According to Cognitive Impairment Status*

| Variable | *B* [95% CI] | β [95% CI] | *R*^2^_adj_ | Δ*R*^2^ | AIC |
| --- | --- | --- | --- | --- | --- |
| NBV |  |  |  |  |  |
| Step 1 |  |  | .22 | .00 | 315.75 |
| Age | –0.15* [–0.20, –0.10] | –0.49* [–0.62, –0.31] |  |  |  |
| Education | –0.01 [–0.26, 0.22] | –0.01 [–0.17, 0.15] |  |  |  |
| Step 2 |  |  | .28 | .06 | 307.39 |
| Age | –0.16* [–0.21, –0.11] | –0.54* [–0.67, –0.38] |  |  |  |
| Education | –0.10 [–0.33, 0.13] | –0.07 [–0.22, 0.08] |  |  |  |
| Impaired | –2.06* [–3.35, –0.81] | –0.55* [–0.86, –0.23] |  |  |  |
| NGMV |  |  |  |  |  |
| Step 1 |  |  | .49 | .00 | 265.32 |
| Age | –0.16* [–0.19, –0.13] | –0.71* [–0.79, –0.59] |  |  |  |
| Education | 0.00 [–0.14, 0.14] | 0.00 [–0.12, 0.14] |  |  |  |
| Step 2 |  |  | .49 | .00 | 266.20 |
| Age | –0.16* [–0.19, –0.13] | –0.72* [–0.80, –0.60] |  |  |  |
| Education | –0.01 [–0.16, 0.14] | –0.01 [–0.14, 0.13] |  |  |  |
| Impaired | –0.41 [–1.17, 0.38] | –0.15 [–0.42, 0.14] |  |  |  |
| NWMV |  |  |  |  |  |
| Step 1 |  |  | –.01 | .00 | 347.12 |
| Age | 0.01 [–0.03, 0.04] | 0.06 [–0.13, 0.23] |  |  |  |
| Education | –0.02 [–0.18, 0.15] | –0.02 [–0.19, 0.15] |  |  |  |
| Step 2 |  |  | .07 | .09 | 337.25 |
| Age | –0.00 [–0.04, 0.03] | –0.01 [–0.19, 0.16] |  |  |  |
| Education | –0.09 [–0.25, 0.07] | –0.09 [–0.26, 0.07] |  |  |  |
| Impaired | –1.65* [–2.60, –0.77] | –0.67* [–0.99, –0.33] |  |  |  |
| CCI |  |  |  |  |  |
| Step 1 |  |  | .00 | .00 | 345.24 |
| Age | 0.00 [ 0.00, 0.01] | 0.11 [–0.09, 0.30] |  |  |  |
| Education | –0.02 [–0.05, 0.02] | –0.08 [–0.23, 0.08] |  |  |  |
| Step 2 |  |  | .03 | .03 | 343.29 |
| Age | 0.00 [–0.01, 0.01] | 0.07 [–0.13, 0.26] |  |  |  |
| Education | –0.03 [–0.06, 0.01] | –0.12 [–0.28, 0.04] |  |  |  |
| Impaired | –0.22 [–0.43, 0.01] | –0.40 [–0.78, 0.00] |  |  |  |

*Note.* All *R*^2^_adj_, Δ*R*^2^, and AIC values are from unstandardised models. GLM = general linear model; *B* = unstandardised coefficient; CI = confidence intervals; β = standardised coefficient; AIC = Akaike information criterion; NBV = normalised brain volume; NGMV = normalised grey matter volume; NWMV = normalised white matter volume; CCI = corpus callosum index; non-impaired = 0, impaired = 1.

*indicates 95% CI does not include 0.

**Appendix D**

**Detailed Results for Brain Volumes as Predictors of Cognitive Subtest Scores**

**Table D.1**

*Detailed Results of the GLMs Examining NBV as a Predictor of Cognitive Subtest Scores*

| Variable | *B* [95% CI] | β [95% CI] | *R*^2^_adj_ | Δ*R*^2^ | AIC |
| --- | --- | --- | --- | --- | --- |
| BVMT–R |  |  |  |  |  |
| Step 1 |  |  | .14 | .00 | 326.79 |
| Age | –0.08 [–0.18, 0.02] | –0.14 [–0.30, 0.03] |  |  |  |
| Education | 1.07* [0.53, 1.54] | 0.37* [0.18, 0.51] |  |  |  |
| Step 2 |  |  | .22 | .08 | 316.07 |
| Age | 0.01 [–0.09, 0.13] | 0.02 [–0.17, 0.21] |  |  |  |
| Education | 1.08* [0.58, 1.52] | 0.37* [0.20, 0.51] |  |  |  |
| NBV | 0.65* [0.26, 1.06] | 0.33* [0.13, 0.51] |  |  |  |
| CVLT–II |  |  |  |  |  |
| Step 1 |  |  | .16 | .00 | 324.24 |
| Age | –0.12 [–0.27, 0.05] | –0.13 [–0.30, 0.04] |  |  |  |
| Education | 1.88* [1.02, 2.71] | 0.40* [0.22, 0.54] |  |  |  |
| Step 2 |  |  | .18 | .02 | 322.60 |
| Age | –0.04 [–0.23, 0.17] | –0.04 [–0.26, 0.16] |  |  |  |
| Education | 1.87* [1.02, 2.71] | 0.40* [0.22, 0.54] |  |  |  |
| NBV | 0.57 [–0.01, 1.15] | 0.18 [–0.01, 0.35] |  |  |  |
| SDMT |  |  |  |  |  |
| Step 1 |  |  | .18 | .00 | 322.31 |
| Age | –0.24* [–0.40, –0.08] | –0.26* [–0.41, –0.08] |  |  |  |
| Education | 1.60* [0.85, 2.47] | 0.34* [0.18, 0.47] |  |  |  |
| Step 2 |  |  | .32 | .14 | 300.67 |
| Age | –0.05 [–0.21, 0.12] | –0.05 [–0.23, 0.12] |  |  |  |
| Education | 1.62* [0.91, 2.37] | 0.34* [0.20, 0.47] |  |  |  |
| NBV | 1.37* [0.77, 1.88] | 0.43* [0.24, 0.57] |  |  |  |

*Note*. All *R*^2^_adj_, Δ*R*^2^, and AIC values are from unstandardised models. GLM = general linear model; NBV = normalised brain volume; *B* = unstandardised coefficient; CI = confidence interval; β standardised coefficient; AIC = Akaike information criterion; BVMT–R = Brief Visuospatial Memory Test-Revised; CVLT–II = California Verbal Learning Test–second edition; SDMT = Symbol Digits Modalities Test.

*indicates 95% CI does not include 0.

**Table D.2**

*Detailed Results of the GLMs Examining NGMV as a Predictor of Cognitive Subtest Scores*

| Variable | *B* [95% CI] | β [95% CI] | *R*^2^_adj_ | Δ*R*^2^ | AIC |
| --- | --- | --- | --- | --- | --- |
| BVMT–R |  |  |  |  |  |
| Step 2 |  |  | .15 | .02 | 326.45 |
| Age | –0.00 [–0.14, 0.15] | –0.01 [–0.24, 0.23] |  |  |  |
| Education | 1.07* [0.55, 1.53] | 0.37* [0.18, 0.51] |  |  |  |
| NGMV | 0.47 [–0.11, 1.17] | 0.18 [–0.05, 0.42] |  |  |  |
| CVLT–II |  |  |  |  |  |
| Step 2 |  |  | .16 | .00 | 325.65 |
| Age | –0.06 [–0.27, 0.16] | –0.06 [–0.30, 0.16] |  |  |  |
| Education | 1.86* [1.02, 2.72] | 0.40* [0.22, 0.54] |  |  |  |
| NGMV | 0.39 [–0.49, 1.22] | 0.09 [–0.11, 0.29] |  |  |  |
| SDMT |  |  |  |  |  |
| Step 2 |  |  | .19 | .02 | 321.08 |
| Age | –0.11 [–0.33, 0.10] | –0.11 [–0.35, 0.10] |  |  |  |
| Education | 1.60* [0.85, 2.46] | 0.34* [0.18, 0.47] |  |  |  |
| NGMV | 0.88 [–0.14, 1.76] | 0.21 [–0.03, 0.41] |  |  |  |

*Note*. Step 1 values are the same as in Supplemental Table 2, and therefore were omitted to avoid repetition of data. All *R*^2^_adj_, Δ*R*^2^, and AIC values are from unstandardised models. GLM = general linear model; NMGV = normalised grey matter volume; *B* = unstandardised coefficient; CI = confidence interval; β = standardised coefficient; AIC = Akaike information criterion; BVMT–R = Brief Visuospatial Memory Test-Revised; CVLT–II = California Verbal Learning Test–second edition; SDMT = Symbol Digits Modalities Test.

*indicates 95% CI does not include 0.

**Table D.3**

*Detailed Results of the GLMs Examining NWMV as a Predictor of Cognitive Subtest Scores*

| Variable | *B* [95% CI] | β [95% CI] | *R*^2^_adj_ | Δ*R*^2^ | AIC |
| --- | --- | --- | --- | --- | --- |
| BVMT–R |  |  |  |  |  |
| Step 2 |  |  | .22 | .08 | 316.73 |
| Age | –0.09 [–0.18, 0.00] | –0.15 [–0.31, 0.01] |  |  |  |
| Education | 1.08* [0.56, 1.54] | 0.37* [0.19, 0.52] |  |  |  |
| NWMV | 0.85* [0.33, 1.33] | 0.28* [0.11, 0.43] |  |  |  |
| CVLT–II |  |  |  |  |  |
| Step 2 |  |  | .18 | .03 | 322.53 |
| Age | –0.13 [–0.28, 0.04] | –0.14 [–0.30, 0.03] |  |  |  |
| Education | 1.88* [1.03, 2.74] | 0.40* [0.22, 0.54] |  |  |  |
| NWMV | 0.76* [0.01, 1.53] | 0.16 [0.00, 0.30] |  |  |  |
| SDMT |  |  |  |  |  |
| Step 2 |  |  | .33 | .15 | 299.15 |
| Age | –0.27* [–0.40, –0.12] | –0.28* [–0.42, –0.12] |  |  |  |
| Education | 1.63* [0.93, 2.36] | 0.35* [0.21, 0.47] |  |  |  |
| NWMV | 1.87* [1.09, 2.63] | 0.39* [0.23, 0.52] |  |  |  |

*Note*. Step 1 values are the same as in Supplemental Table 2, and therefore were omitted to avoid repetition of data. All *R*^2^_adj_, Δ*R*^2^, and AIC values are from unstandardised models. GLM = general linear model; NMWV = normalised white matter volume; *B* = unstandardised coefficient; CI = confidence interval; β = standardised coefficient; AIC = Akaike information criterion; BVMT–R = Brief Visuospatial Memory Test-Revised; CVLT–II = California Verbal Learning Test–second edition; SDMT = Symbol Digits Modalities Test.

*indicates 95% CI does not include 0.

**Table D.4**

*Detailed Results of the GLMs Examining CCI as a Predictor of Cognitive Subtest Scores*

| Variable | *B* [95% CI] | β [95% CI] | *R*^2^_adj_ | Δ*R*^2^ | AIC |
| --- | --- | --- | --- | --- | --- |
| BVMT–R |  |  |  |  |  |
| Step 2 |  |  | .15 | .02 | 326.30 |
| Age | –0.09 [–0.18, 0.01] | –0.15 [–0.31, 0.01] |  |  |  |
| Education | 1.10* [0.55, 1.58] | 0.38* [0.19, 0.52] |  |  |  |
| CCI | 1.77 [–0.38, 3.88] | 0.14 [–0.03, 0.28] |  |  |  |
| CVLT–II |  |  |  |  |  |
| Step 2 |  |  | .16 | .00 | 325.93 |
| Age | –0.12 [–0.27, 0.04] | –0.13 [–0.30, 0.04] |  |  |  |
| Education | 1.88* [1.02, 2.75] | 0.40* [0.22, 0.55] |  |  |  |
| CCI | 1.01 [–2.42, 4.27] | 0.05 [–0.12, 0.19] |  |  |  |
| SDMT |  |  |  |  |  |
| Step 2 |  |  | .21 | .04 | 318.44 |
| Age | –0.26* [–0.41, –0.11] | –0.28* [–0.43, –0.11] |  |  |  |
| Education | 1.67* [0.89, 2.51] | 0.36* [0.19, 0.49] |  |  |  |
| CCI | 4.20* [0.92, 7.53] | 0.20* [0.04, 0.34] |  |  |  |

*Note*. Step 1 values are the same as in Supplemental Table 2, and therefore were omitted to avoid repetition of data. All *R*^2^_adj_, Δ*R*^2^, and AIC values are from unstandardised models. GLM = general linear model; CCI = corpus callosum index; *B* = unstandardised coefficient; CI = confidence interval; β = standardised coefficient; AIC = Akaike information criterion; BVMT–R = Brief Visuospatial Memory Test-Revised; CVLT–II = California Verbal Learning Test–second edition; SDMT = Symbol Digits Modalities Test.

*indicates 95% CI does not include 0.

**Appendix E**

**Detailed Results for Additional Cognitive Tests**

**Table E.1**

*Detailed Results of the GLMs Examining Neuroimaging Markers as Predictors of Additional Cognitive Tests*

| Model | *B* [95% CI] | β [95% CI] | *R*^2^_adj_ | Δ*R*^2^ | AIC |
| --- | --- | --- | --- | --- | --- |
| BVMT–R Delayed Recall |  |  |  |  |  |
| NBV | 0.22* [0.04, 0.43] | 0.31* [0.04, 0.54] | .20 | .07 | 76.25 |
| NGMV | 0.16 [–0.08, 0.47] | 0.16 [–0.10, 0.41] | .03 | .01 | 81.36 |
| NWMV | 0.30* [0.07, 0.53] | 0.26* [0.06, 0.44] | .13 | .07 | 78.48 |
| CCI | 0.48 [–1.40, 1.38] | 0.10 [–0.09, 0.27] | –.01 | .01 | 82.60 |
| CVLT–II Long Delay |  |  |  |  |  |
| NBV | 0.08 [–0.08, 0.26] | 0.09 [–0.09, 0.27] | .02 | .01 | 81.77 |
| NGMV | 0.15 [–0.11, 0.39] | 0.12 [–0.08, 0.32] | .02 | .01 | 81.63 |
| NWMV | 0.05 [–0.20, 0.28] | 0.03 [–0.13, 0.19] | .02 | .00 | 81.66 |
| CCI | –0.29 [–1.39, 0.75] | –0.05 [–0.21, 0.12] | .05 | .00 | 80.98 |
| DSF |  |  |  |  |  |
| NBV | 0.00 [–0.15, 0.14] | 0.00 [–0.24, 0.24] | .12 | .00 | 78.77 |
| NGMV | –0.01 [–0.23, 0.17] | –0.02 [–0.27, 0.22] | .11 | .00 | 79.17 |
| NWMV | 0.01 [–0.17, 0.19] | 0.01 [–0.19, 0.20] | .11 | .00 | 79.01 |
| CCI | –0.10 [–0.92, 0.70] | –0.03 [–0.23, 0.17] | .13 | .00 | 78.45 |
| DSB |  |  |  |  |  |
| NBV | 0.04 [–0.08, 0.16] | 0.07 [–0.15, 0.28] | –.11 | .00 | 85.00 |
| NGMV | 0.03 [–0.16, 0.20] | 0.05 [–0.22, 0.27] | –.11 | .00 | 85.17 |
| NWMV | 0.05 [–0.11, 0.24] | 0.05 [–0.14, 0.24] | –.11 | .00 | 85.10 |
| CCI | 0.11 [–0.67, 0.72] | 0.03 [–0.15, 0.21] | –.11 | .00 | 85.23 |
| TMT–A |  |  |  |  |  |
| NBV | –0.80* [–1.58, –0.02] | –0.31 [–0.55, 0.01] | .15 | .06 | 77.84 |
| NGMV | –0.78 [–1.82, 0.24] | –0.23 [–0.48, 0.08] | .08 | .02 | 79.96 |
| NWMV | –0.78 [–1.64, 0.07] | –0.19 [–0.36, 0.02] | .18 | .03 | 77.07 |
| CCI | –3.11 [–6.83, 1.19] | –0.18 [–0.37, 0.07] | .21 | .03 | 75.81 |
| TMT–B |  |  |  |  |  |
| NBV | –1.60 [–3.60, 0.65] | –0.17 [–0.43, 0.05] | .03 | .02 | 81.43 |
| NGMV | –3.75* [–6.98, –0.16] | –0.31* [–0.60, –0.01] | .03 | .05 | 81.49 |
| NWMV | 0.02 [–2.36, 2.60] | 0.00 [–0.18, 0.17] | .03 | .00 | 81.40 |
| CCI | 3.16 [–7.04, 17.47] | 0.05 [–0.16, 0.22] | .09 | .00 | 79.72 |

**Table E.1** (continued).

| Model | *B* [95% CI] | β [95% CI] | *R*^2^_adj_ | Δ*R*^2^ | AIC |
| --- | --- | --- | --- | --- | --- |
| Logical Memory I |  |  |  |  |  |
| NBV | 0.38 [–0.13, 0.84] | 0.19 [–0.06, 0.43] | .22 | .02 | 75.49 |
| NGMV | 0.57 [–0.21, 1.30] | 0.22 [–0.08, 0.48] | .21 | .02 | 76.05 |
| NWMV | 0.28 [–0.38, 0.82] | 0.09 [–0.10, 0.28] | .21 | .01 | 75.93 |
| CCI | –0.01 [–3.12, 2.60] | 0.00 [–0.21, 0.21] | .24 | .00 | 75.02 |
| Logical Memory II |  |  |  |  |  |
| NBV | 0.59* [0.04, 1.08] | 0.30* [0.02, 0.54] | .05 | .06 | 80.94 |
| NGMV | 0.67 [–0.14, 1.35] | 0.26 [–0.06, 0.51] | .08 | .03 | 80.10 |
| NWMV | 0.59 [–0.09, 1.20] | 0.19 [–0.03, 0.39] | –.04 | .03 | 83.38 |
| CCI | 1.12 [–2.00, 3.86] | 0.09 [–0.15, 0.30] | –.04 | .01 | 83.31 |
| CFT |  |  |  |  |  |
| NBV | 0.40* [0.06, 0.69] | 0.28* [0.04, 0.50] | .00 | .05 | 82.10 |
| NGMV | 0.25 [–0.34, 0.75] | 0.13 [–0.18, 0.41] | .00 | .01 | 82.07 |
| NWMV | 0.49* [0.05, 0.88] | 0.22* [0.02, 0.39] | .05 | .05 | 81.03 |
| CCI | 1.17 [–0.50, 2.98] | 0.12 [–0.05, 0.30] | .05 | .01 | 80.77 |
| COWAT |  |  |  |  |  |
| NBV | 0.48 [–0.23, 1.12] | 0.15 [–0.07, 0.35] | –.01 | .01 | 82.67 |
| NGMV | 0.60 [–0.52, 1.57] | 0.14 [–0.12, 0.38] | .07 | .01 | 80.20 |
| NWMV | 0.40 [–0.57, 1.42] | 0.08 [–0.11, 0.27] | .02 | .01 | 81.77 |
| CCI | 2.32 [–1.51, 6.84] | 0.11 [–0.08, 0.29] | .02 | .01 | 81.81 |
| SYDBAT |  |  |  |  |  |
| NBV | 0.11 [–0.19, 0.47] | 0.17 [–0.35, 0.58] | .04 | .02 | 81.09 |
| NGMV | 0.11 [–0.17, 0.50] | 0.14 [–0.26, 0.51] | .02 | .01 | 80.75 |
| NWMV | 0.11 [–0.27, 0.70] | 0.11 [–0.32, 0.50] | .06 | .01 | 81.09 |
| CCI | 0.17 [–1.10, 2.06] | 0.04 [–0.30, 0.35] | .02 | .00 | 81.64 |
| BNT |  |  |  |  |  |
| NBV | 0.19 [–0.29, 0.71] | 0.11 [–0.14, 0.44] | .17 | .01 | 77.20 |
| NGMV | 0.05 [–0.85, 0.63] | 0.02 [–0.26, 0.38] | .24 | .00 | 74.94 |
| NWMV | 0.28 [–0.25, 0.92] | 0.10 [–0.10, 0.34] | .17 | .00 | 77.42 |
| CCI | 0.63 [–1.77, 4.19] | 0.06 [–0.23, 0.27] | .16 | .00 | 77.50 |
| VST |  |  |  |  |  |
| NBV | –0.51 [–1.21, 0.04] | –0.22 [–0.44, 0.01] | .25 | .04 | 74.47 |
| NGMV | –0.06 [–0.94, 0.63] | –0.02 [–0.28, 0.22] | .26 | .00 | 74.20 |
| NWMV | –0.90 [–1.86, 0.01] | –0.25 [–0.47, 0.02] | .23 | .06 | 75.10 |
| CCI | –3.04 [–7.16, 0.38] | –0.20 [–0.41, 0.04] | .24 | .04 | 74.96 |

*Note*. Age and education were included in all models. All *R*^2^_adj_, Δ*R*^2^, and AIC values are from unstandardised models. GLM = general linear model; *B* = unstandardised coefficient; CI = confidence intervals; β = standardised coefficient; AIC = Akaike information criterion; BVMT–R = Brief Visuospatial Memory Test–Revised; NBV = normalised brain volume; NGMV = normalised grey matter volume; NWMV = normalised white matter volume; CCI = corpus callosum index; CVLT–II = California Verbal Learning Test–second edition; DSF = Digit Span Forwards; DSB = Digit Span Backwards; TMT–A = Trail Making Test part A; TMT–B = Trail Making Test part B; CFT = Categorical Fluency Test; COWAT = Controlled Oral Word Association Test; SYDBAT = Sydney Language Battery; BNT = Boston Naming Test; VST = Victoria Stroop Test.

*indicates FCR-adjusted CI does not include zero.

**eReferences**

1. Benedict RHB. Brief Visuospatial Memory Test–Revised: Professional manual. Psychological Assessment Resources; 1997.

2. Benedict RHB, Schretlen D, Groninger L, Dobraski M, Shpritz B. Revision of the Brief Visuospatial Memory Test: Studies of normal performance, reliability, and validity. Psychol Assess. US: American Psychological Association; 1996;8:145–53. https://doi.org/10.1037/1040-3590.8.2.145

3. Delis DC, Kramer JH, Kaplan E, Ober BA. California Verbal Learning Test–Second Edition (CVLT-II): Adult version manual. The Psychological Corporation; 2000.

4. Wechsler D. Wechsler Adult Intelligence Scale–Fourth Edition (WAIS–IV): Technical and interpretive manual. Pearson; 2008.

5. Reitan RM. Validity of the Trail Making Test as an Indicator of Organic Brain Damage. Percept Mot Skills. SAGE Publications Inc; 1958;8:271–6. https://doi.org/10.2466/pms.1958.8.3.271

6. Tombaugh TN. Trail Making Test A and B: normative data stratified by age and education. Arch Clin Neuropsychol Off J Natl Acad Neuropsychol. 2004;19:203–14. https://doi.org/10.1016/S0887-6177(03)00039-8

7. Wechsler D. Wechsler Memory Scale–Fourth Edition (WMS-IV). Psychological Corporation; 2009.

8. Benton AL, Hamsher KDS, Sivan AB. Multilingual aphasia examination. 3rd ed. AJA Associates; 1994.

9. Tombaugh TN, Hubiey AM. The 60-item boston naming test: Norms for cognitively intact adults aged 25 to 88 years. J Clin Exp Neuropsychol. Routledge; 1997;19:922–32. https://doi.org/10.1080/01688639708403773

10. Lezak MD, Howieson DB, Bigler ED, Tranel D. Neuropsychological assessment. 5th ed. Oxford University Press; 2012.

11. Strauss E, Sherman EMS, Spreen O. A compendium of neuropsychological tests: Administration, norms, and commentary. 3rd ed. Oxford University Press; 2006.

12. Rees L, Tombaugh TN, Kozak J. Normative data for two verbal fluency tests (FAS and “Animals”) for 1300 cognitively intact individuals aged 16–90 years. Arch Clin Neuropsychol. 1998;13:101. https://doi.org/10.1093/arclin/13.1.101

13. Savage S, Hsieh S, Leslie F, Foxe D, Piguet O, Hodges JR. Distinguishing Subtypes in Primary Progressive Aphasia: Application of the Sydney Language Battery. Dement Geriatr Cogn Disord. 2013;35:208–18. https://doi.org/10.1159/000346389

14. Kaplan E, Goodglass H, Weintraub S. Boston naming test. Lea & Febiger; 1983.

15. Troyer AK, Leach L, Strauss E. Victoria Stroop Test. Psychological Assessment Resources; 1996.

16. R Core Team. R: A language and environment for statistical computing [Internet]. R Foundation for Statistical Computing; 2024. https://www.R-project.org/

17. Hond AAH de, Steyerberg EW, Calster B van. Interpreting area under the receiver operating characteristic curve. Lancet Digit Health. Elsevier; 2022;4:e853–5. https://doi.org/10.1016/S2589-7500(22)00188-1

18. Roche A, Maréchal B, Kober T, Krueger G, Hagmann P, Maeder P, et al. Assessing Brain Volumes Using MorphoBox Prototype. Magnetom Flash [Internet]. Siemens Healthineers; 2017;68. https://cdn0.scrvt.com/39b415fb07de4d9656c7b516d8e2d907/1800000003913239/f9d95e26c89c/magnetom-flash-68_assessing-brain-volumes-using-morphobox-prototype_roche_1800000003913239.pdf

19. Sammut-Powell C, Reynard C, Allen J, McDermott J, Braybrook J, Parisi R, et al. Examining the effect of evaluation sample size on the sensitivity and specificity of COVID-19 diagnostic tests in practice: a simulation study. Diagn Progn Res. 2022;6:12. https://doi.org/10.1186/s41512-021-00116-4

20. Benjamini Y, Yekutieli D. False Discovery Rate–Adjusted Multiple Confidence Intervals for Selected Parameters. J Am Stat Assoc. Taylor & Francis; 2005;100:71–81. https://doi.org/10.1198/016214504000001907
